# Microbial Diversity and Metabolite Profile of Fermenting Millet in the Production of *Hausa koko*, a Ghanaian Fermented Cereal Porridge

**DOI:** 10.3389/fmicb.2021.681983

**Published:** 2021-08-04

**Authors:** Amy Atter, Maria Diaz, Kwaku Tano-Debrah, Angela Parry-Hanson Kunadu, Melinda J. Mayer, Ian J. Colquhoun, Dennis Sandris Nielsen, David Baker, Arjan Narbad, Wisdom Amoa-Awua

**Affiliations:** ^1^Food Microbiology and Mushroom Research Division, CSIR-Food Research Institute, Accra, Ghana; ^2^Department of Nutrition and Food Science, University of Ghana, Accra, Ghana; ^3^Food and Health Institute Strategic Programme, Quadram Institute Bioscience, Norwich Research Park, Norwich, United Kingdom; ^4^Gut Microbes and Health Institute Strategic Programme, Quadram Institute Bioscience, Norwich Research Park, Norwich, United Kingdom; ^5^Analytical Sciences Unit, Quadram Institute Bioscience, Norwich Research Park, Norwich, United Kingdom; ^6^Department of Food Science, Section for Food Microbiology and Fermentation, University of Copenhagen, Copenhagen, Denmark; ^7^Quadram Institute Bioscience, Norwich Research Park, Norwich, United Kingdom; ^8^Department of Agro-Processing Technology and Food Bio-Sciences, CSIR College of Science and Technology, Accra, Ghana

**Keywords:** fermented cereal, *Hausa koko*, Africa, metabolomics, bacteria, fungi, millet

## Abstract

*Hausa koko* is an indigenous porridge processed from millet in Ghana. The process involves fermentation stages, giving the characteristic organoleptic properties of the product that is produced largely at a small-scale household level and sold as a street food. Like many other indigenous foods, quality control is problematic and depends on the skills of the processor. In order to improve the quality of the product and standardize the process for large-scale production, we need a deeper understanding of the microbial processes. The aim of this study is to investigate the microbial community involved in the production of this traditional millet porridge and the metabolites produced during processing. High-throughput amplicon sequencing was used to identify the bacterial (16S rRNA V4 hypervariable region) and fungal [Intergenic Transcribed Spacer (ITS)] communities associated with the fermentation, while nuclear magnetic resonance (NMR) was used for metabolite profiling. The bacterial community diversity was reduced during the fermentation processes with an increase and predominance of lactobacilli. Other dominant bacteria in the fermentation included *Pediococcus*, *Weissella*, *Lactococcus*, *Streptococcus*, *Leuconostoc*, and *Acetobacter.* The species *Limosilactobacillus fermentum* and *Ligilactobacillus salivarius* accounted for some of the diversities within and between fermentation time points and processors. The fungal community was dominated by the genus *Saccharomyces*. Other genera such as *Pichia*, *Candida*, *Kluyveromyces*, *Nakaseomyces*, *Torulaspora*, and *Cyberlindnera* were also classified. The species *Saccharomyces cerevisiae*, *Stachybotrys sansevieriae*, *Malassezia restricta*, *Cyberlindnera fabianii*, and *Kluyveromyces marxianus* accounted for some of the diversities within some fermentation time points. The species *S. sansevieria* and *M. restricta* may have been reported for the first time in cereal fermentation. This is the most diverse microbial community reported in *Hausa koko*. In this study, we could identify and quantify 33 key different metabolites produced by the interactions of the microbial communities with the millet, composed of organic compounds, sugars, amino acids and intermediary compounds, and other key fermentation compounds. An increase in the concentration of organic acids in parallel with the reduction of sugars occurred during the fermentation process while an initial increase of amino acids followed by a decrease in later fermentation steps was observed.

## Introduction

Porridges produced from fermented cereals such as maize, millet, and sorghum are essential diets in many parts of Africa, where they are used mostly as staple, weaning, or complementary foods, providing necessary nutrients (Omemu et al., 2007; Omemu and Omeike, 2010; Owusu-Kwarteng et al., 2010b). In Ghana, fermented thin cereal porridges are called *koko* and are usually eaten at breakfast. Fermented stiff cereal porridges are eaten as main meals mostly at lunch or dinner and include *kenkey*, *banku*, and *tuo zaafi.* One of the most popular of the Ghanaian thin porridges is *Hausa koko*, a spicy, smooth, and free-flowing fermented millet porridge that is mostly produced and sold as street food (Mensah et al., 2002; Haleegoah et al., 2016).

**FIGURE 1:**
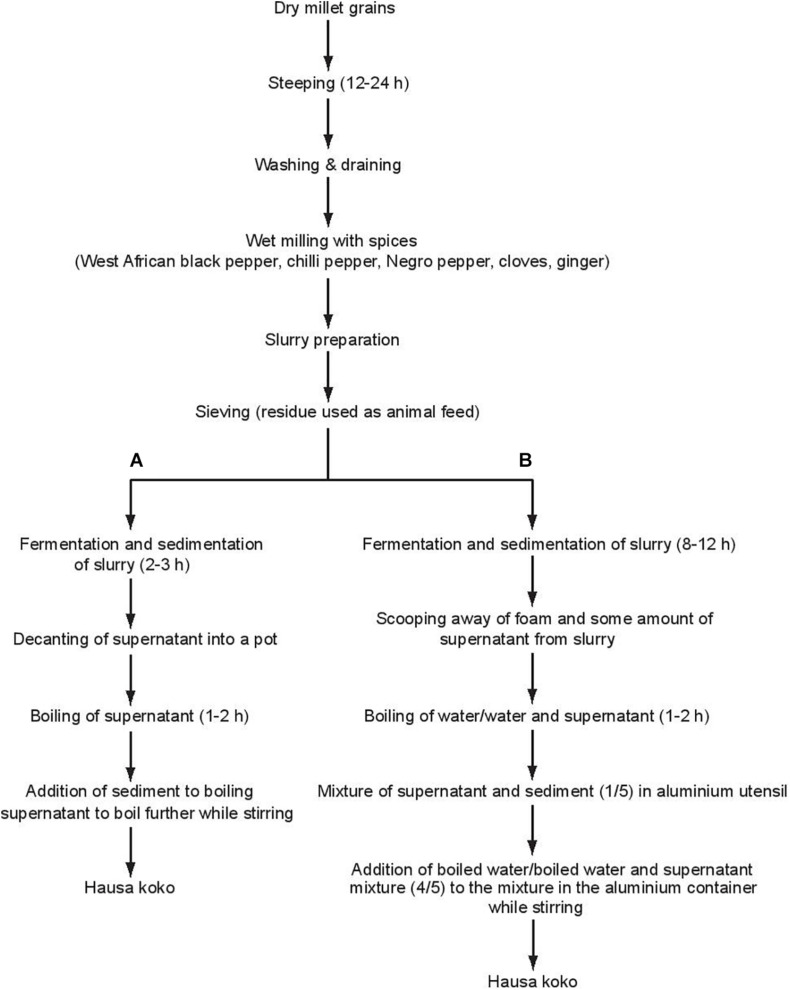
Flow diagram of *Hausa koko* production process. **(A)** Process described by Lei and Jakobson (2004). **(B)** Process described in the current work.

*Hausa koko* is produced by traditional food processors as a micro or cottage industry in mainly home-based operations using the two main indigenous methods shown in Figure 1. Lei and Jakobsen (2004) described and studied Process A while Process B is more commonly used and is the subject of the present investigation. The processes in *Hausa koko* production involve initial steeping of millet grains for 12–24 h, during which fermentation starts. The steeped grains are then washed and milled together with different spices in a plate mill. The resulting flour is mixed with water to prepare a slurry, which is then sieved using a cheese cloth. The slurry is allowed to ferment spontaneously for 8–12 h and becomes sour. During the fermentation of the slurry, it settles into a sediment and a supernatant with a foam sitting on top. The foam is scooped off and discarded, while the supernatant may be consumed in its raw state as *koko sour water* for medicinal purposes, especially for treatment of diarrhea. Indeed, Lei et al. (2006) have confirmed that *koko sour water* has natural probiotic properties. To prepare *Hausa koko*, most processors boil four volumes of water, and while stirring the fermented slurry (the supernatant and sediment together representing one volume), slowly pour in the hot boiled water to obtain the smooth gel-like porridge. A few processors, however, add water to the supernatant and boil the diluted supernatant. The hot diluted supernatant is then poured slowly into the sediment while stirring continuously to obtain *Hausa koko*. *Hausa koko* is sweetened with sugar before consumption and many consumers may add milk to the thin porridge or even roasted groundnuts. Traditionally, *Hausa koko* may be eaten with *koose*, a fried cowpea doughnut or *masa*, a fried sour millet, maize, or rice doughnut; however, *Hausa koko* is often eaten with bread.

Only a few studies have been carried out to study the microbiology of *Hausa koko* processing, notably the work by Lei and Jakobsen (2004). They reported the spontaneous fermentation to be dominated by *Weissella confusa* and *Lactobacillus* (now *Limosilactobacillus*) *fermentum*, showing a pronounced taxonomic biodiversity at sub-species level between stages within the production as well as between production sites. Other species reported in *koko* sour water by Lei and Jakobsen (2004) were *Lactobacillus* (now *Ligilactobacillus*) *salivarius*, *Pediococcus pentosaceus*, *Pediococcus acidilactici*, and *Lactobacillus* (now *Lactiplantibacillus*) *paraplantarum*, and live LAB content of *koko* sour water has been estimated as c. 10^8^ cells per ml.

Consumption of *Hausa koko* gives some of the benefits of fermentation. Fermentation of cereals is reported to reduce the levels of some anti-nutrients including tannins, polyphenols, and phytates, some of which can inhibit amylolysis and proteolysis and sequester proteins and valuable minerals in complexes; microbial enzymatic activity can disrupt these complexes and improve the bioavailability of minerals such as iron, calcium, zinc, and phosphorus (Sharma and Kapoor, 1996; Sindhu and Khetarpaul, 2001; Blandino et al., 2003). Fermentation also enhances the nutritional and sensory qualities as well as shelf life of the products, among many other benefits (Holzapfel, 2002; Ibnouf, 2012; Arena et al., 2014). Cereal fermented foods are also reported to provide health benefits such as blood-lowering effects, inhibition of allergies, antimicrobial effects, and control of diarrhea through probiotic mechanisms (Lei et al., 2006; Vasiljevic and Shah, 2008; Das et al., 2012; Zannini et al., 2012; Wang et al., 2014; Ray et al., 2016).

In the production of indigenous African foods, fermentations are carried out mostly spontaneously, i.e., without the use of a traditional inoculum or a starter culture. However, the products attain a low pH. In spite of this, the microbiological quality of *Hausa koko* and other porridges seem unpredictable. According to Yeleliere et al. (2017) indigenous Ghanaian cereal porridges can be contaminated with pathogens including *Bacillus cereus*, *Staphylococcus aureus*, and *Enterobacteriaceae*, which are significant in terms of food safety.

In the past few decades, several successful attempts have been made to upgrade the technologies used in the production of some indigenous African foods, including fermented products. Many of these are now produced by small- and medium-scale enterprise (SME) as convenience foods for both local and foreign markets targeting Africans. Attempts have also been made to use starter cultures at the SME level to gain a greater control over the process, but this is yet to be widely adopted by industry. The use of starter cultures, however, is seen as a prerequisite for standardizing the sensory quality of the products and improving their safety. In Ghana, the use of starter culture to produce *kenkey*, a cooked fermented maize dumpling, was demonstrated at an upgraded traditional food processing facility, but could not be sustained at the facility by the plant owner (Halm et al., 1996; Amoa-Awua et al., 2007). With regard to *Hausa koko*, an in-depth understanding of the microbial community involved in its production is required, if a suitable starter culture is to be developed for the production of *Hausa koko* by the Ghanaian food industry as a high-quality convenience food.

Porridges made from fermented cereals are also reported to have micronutrient deficiencies, hence require some fortification (Owusu-Kwarteng et al., 2010a; Tano-Debrah et al., 2019). In view of this, a food supplement known as KOKO plus was used to improve the nutritional profile of *koko* produced from fermented maize dough, while *Hausa koko* from pearl millet was fortified with soybean for complementary feeding for infants and children (Owusu-Kwarteng et al., 2010a; Tano-Debrah et al., 2019). Unfortunately, there is no information available on the nutritional profile and other metabolites produced during the processing of millet into *Hausa koko.*

This study was carried out to determine the diversity of microorganisms involved in the processing of millet into *Hausa koko* following 15 small-scale processors located in six regions of Ghana, using high-throughput sequencing technology. It also sought to determine the metabolites produced during the process.

## Materials and Methods

### Sampling

Samples of millet-based *Hausa koko* taken during the main steps involved in its production were collected from 15 small-scale processing sites. The sites were located in six regions of Ghana, distributed in three different geographical belts – northern, middle, and southern belts. The sites were distributed as follows: Northern region (Northern belt) – Tamale Central (TAC), Tamale Kalariga (TAK), and Tamale Dabokpa (TAD); Bono East Region (Middle belt) – Techiman Diasempa (TED), Techiman Abourso (TEA), Techiman Pomaakrom (TEP), and Techiman Kenten (TEK); Bono Region (Middle belt) – Sunyani (SUN); Central Region (Southern belt) – Mankessim (MAN) and Winneba (WIN); Eastern Region (Southern Belt) – Dodowa (DOD); Greater Accra Region (Southern belt) – Accra Ashaiman-Tulaku (AAT), Accra Madina Zongo (AMZ), Accra Ashaley Botwe (AAB), and Accra Ashaiman-Fafraha (AAF). The following samples were collected during processing at sites: dry millet (D), 12-h fermented millet (12 h), 24-h fermented millet (24 h), milled millet with spices (M), supernatant of slurry (Su), sediment of slurry (Sd), and *Hausa koko* (K). About 500 g of each sample was aseptically collected in duplicates into sterile bags and containers and transported to the CSIR-Food Research Institute (FRI) Microbiology laboratory under cold storage, where they were preserved at -20°C. The samples were later transported under cold conditions (frozen with iced packs) to the Quadram Institute Bioscience (QIB, Norwich, United Kingdom), where they were also preserved at −20°C and processed for further analysis.

### Total Microbial DNA Extraction From Fermented Samples

Microbial DNA from the fermented samples was extracted according to Diaz et al. (2019), with minor modifications. First, 20 g of each sample was homogenized with 10 ml of ice-cold sterile ultrapure H_2_O by vortexing and centrifuged (Eppendorf 5810R, Germany) for 1 min at 800 × *g* at 4°C to remove the solid particles of the sample. This process was repeated twice, and the resulting supernatants were pooled together and centrifuged at 3,000 × *g* at 4°C for 20 min to harvest the cells. The pellets were washed three times with 1 ml of phosphate buffered saline (PBS) and centrifuged at 14,000 × *g* for 2 min. Microbial DNA was extracted from the pellets using the FastDNA spin kit for soil (MP Biomedicals, United States). All steps were performed following the manufacturer’s instructions, with cell lysis performed mechanically using a FastPrep-24 instrument (MP Biomedicals, United Kingdom) for 60 s at a speed of 6.5 m/s. The process was repeated three times with samples kept on ice for 5 min between each homogenization. DNA was eluted in 50 μl of DES (DNase/Pyrogen free water) pre-warmed at 55°C and quantified using the Qubit 3.0 fluorometer (Invitrogen, Malaysia) using the Qubit dsDNA Broad Range (BR) Assay kit or the Qubit dsDNA High Sensitivity (HS) Assay kit (Invitrogen), depending on the DNA concentration of the sample.

### 16S rRNA Amplicon Sequencing

Bacterial diversity was analyzed by 16S rRNA high-throughput amplicon sequencing. Amplification and sequencing were performed by Novogene (HK) Company Limited (Hong Kong) as follows. The V4 hypervariable region of the 16S rRNA gene was amplified by PCR using specific primers 515F and 806R (Caporaso et al., 2011) and the Phusion High-Fidelity PCR master mix (New England Biolabs, United States), following the manufacturer’s instructions. The amplicons were used to generate libraries using the Illumina NEBNext Ultra II DNA Library Prep Kit (New England Biolabs, United Kingdom) and then sequenced using paired-end Illumina sequencing (2 × 250 bp) on the HiSeq 2500 platform (Illumina, United States).

### ITS Amplicon Sequencing

Fungal diversity was analyzed by high-throughput amplicon sequencing of the internal transcribed spacer (ITS). For this analysis, a subset of samples obtained from five processors were included (MAN, SUN, TAD, TEK, and WIN). Amplification and sequencing were performed at QIB (Norwich, United Kingdom). The ITS region was amplified by using primers ITS1F (5′-CTTGGTCATTTAGAGGAAGTAA-3′) (Jackson et al., 1999) and ITS2 (5′-GCTGCGTTCTTCATCGATGC-3′) (White et al., 1990) and the KAPA2G Robust HotStart PCR Kit (Sigma). Amplification was performed at 95°C for 5 min, 30 cycles of 95°C for 30 s, 55°C for 30 s, and 72°C for 30 s followed by a final 72°C for 5 min. PCR products were purified using KAPA Pure Beads (Roche) and used to generate a library using the KAPA2G Robust HotStart PCR Kit and the Nextera XT Index Kit v2 index primers (Illumina) following the manufacturer’s instructions. The libraries were sequenced on an Illumina MiSeq instrument using MiSeq^®^ Reagent Kit v3 (Illumina) following the Illumina denaturation and loading recommendations.

### Metabolite Analysis and Quantification

Metabolites from samples at selected stages (D, 12 h, 24 h, M, Su, and K) were analyzed by ^1^H-nuclear magnetic resonance spectroscopy (NMR). The solid samples were ground into fine flour with a laboratory mortar and pestle, and 1 g of the product was mixed with 4 ml of ultra-pure H_2_O by vortexing for 30 s. Liquid and semi-liquid samples were homogenized by vortexing, and 5 ml was used for further analysis. All samples were centrifuged at 2,000 × *g* for 5 min at 4°C and 400 μl of the supernatant was collected and mixed with 400 μl of NMR buffer (4.2 g NaH_2_PO_4_.H_2_O, 3.3 g K_2_HPO_4_, 17.2 mg of Na_3_PO_4_, and 20 mg NaN_3_ in 100 μl of 100 mM EDTA in H_2_O). Six hundred microliters of the mixture was transferred into 5-mm NMR borosilicate glass NMR tubes (Wilmad, Vineland, NJ, United States) and run on a 600-MHz AVANCE^TM^ spectrometer (Bruker, Billerica, MA, United States) with cryoprobe. The recorded spectra were transformed with a 0.3-Hz line broadening, and manually phased, baseline-corrected, and referenced by setting the TSP methyl signal to 0 ppm using the TopSpin software. Metabolites were identified and quantified by computer-assisted manual fitting with Chenomx NMR suite v 8.12 (Chenomx, Edmonton, AB, Canada), using Chenomx 600 MHz HMDB Compounds library.

### Bioinformatic and Statistical Analysis

Bacterial and fungal diversity were analyzed using the Quantitative Insights Into Microbial Ecology 2 (QIIME2) 2020.8 software (Bolyen et al., 2019). The demultiplexed paired-end reads were filtered to remove substitution and chimera errors and merged using DADA2. Bacterial taxonomic assignment was performed at 97% similarity using a Naive Bayes classifier trained on the Silva version 138.1 99% operational taxonomic unit (OTU) database, where the sequences have been trimmed to only include 250 bases from the V4 region bound by the 515 F/806 R primer pair. For fungal characterization, primer and adapter sequences were removed from the reads. Analysis was carried out with the forward reads only to avoid bias caused by amplicons of larger length (i.e., larger than ca. 500 bp) that could not be merged (Taylor et al., 2016). The demultiplexed single-end reads were filtered to remove substitution and chimera errors and merged using DADA2 and taxonomic assignment was performed at 97% using a Naïve Bayes classifier trained on the UNITE Community (2019) fungal classifier version 8_99. Bacterial and fungal alpha diversity was analyzed using observed OTUs, Shannon, Pielou’s evenness, and Faith’s phylogenetic diversity indexes. Rarefaction curves were computed using observed OTUs. Significant differences in alpha diversity were calculated using the alpha-group-significance script in QIIME2, which performs the Kruskal–Wallis test. Jaccard, unweighted, and weighted UniFrac distances were used to generate beta diversity PCoA biplots, which were visualized using the Emperor tool. Differences in beta diversity between groups were analyzed using PERMANOVA including pairwise test (Anderson, 2017). Significant differences in the bacterial community structure among the groups were evaluated by Analysis of Composition of Microbiomes (ANCOM) (Mandal et al., 2015). For metabolites, statistical analysis using two-way ANOVA with Tukey’s multiple comparisons test was applied using GraphPad Prism version 8.4.3. A *p*-value ≤ 0.05 was considered statistically significant.

## Results

### Bacterial Diversity

**FIGURE 2:**
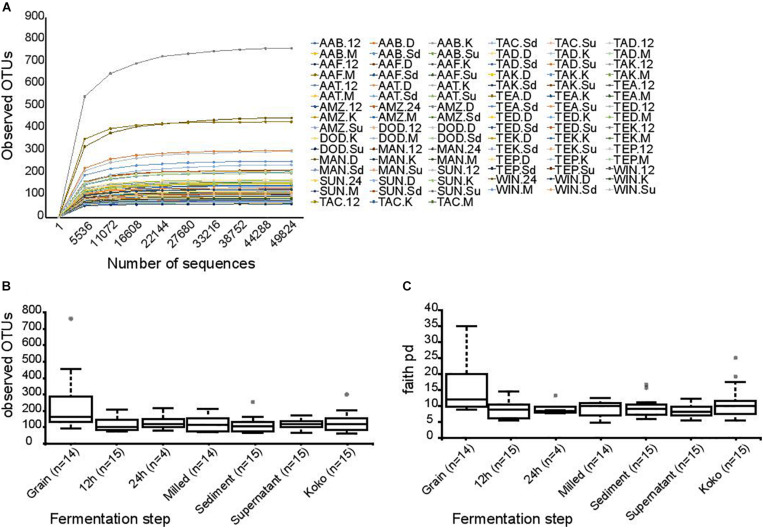
Bacterial alpha diversity indexes. **(A)** Rarefaction plots of observed OTUs depending on the number of sequences. Each curve represents a sample. Observed OTUs **(B)** and Faith’s phylogeny **(C)** diversity box plots for each fermentation step. Dots represent outliers.

DNA sequencing of the V4 amplicons by the Illumina HiSeq platform resulted in 8,361,221 paired-end sequence reads with an average of 89,905.60 ± 5932.20 sequences per sample. Of these, 18.5% were discarded due to poor quality, reads not merging, or after being identified as chimeras; as a result of these steps, 6,813,680 high-quality sequences were retained and analyzed, with an average of 73,265.37 ± 6301.12 sequences per sample. Distribution of reads per sample can be found in Supplementary Table 1. Background DNA was removed by filtering out sequences assigned to chloroplast and mitochondrial taxonomic groups. Data were rarefied to 49,824 sequences per sample to avoid bias. As shown in Figure 2A, the rarefaction curves for the observed OTUs were enough to capture the diversities within all the samples.

Alpha diversity indexes (observed OTUs, Shannon, Faith’s phylogenetic diversity, and Pielou’s evenness) were compared based on the fermentation stages. Significant differences were detected between the observed OTUs at the various time points (*p*-value = 0.026), a reduction in the observed OTUs was detected between the grain samples and the 12-h fermentation, and the lower diversity was maintained during the production stages (*p*-values: vs. 12 h = 0.003; vs. milled = 0.004; vs. supernatant = 0.004; vs. sediment = 0.001; vs. koko = 0.012) (Figure 2B). Significant differences in the alpha diversity were also found between grain samples and other fermentation stages when using Faith’s phylogeny diversity index (Figure 2C) (*p*-value = 0.030 for all groups, *p*-values of grain vs.: 12 h = 0.005; 24 h = 0.033; milled = 0.021; supernatant = 0.002; sediment = 0.005; koko = 0.036). No statistical differences were observed when evenness and Shannon indexes were used.

**FIGURE 3:**
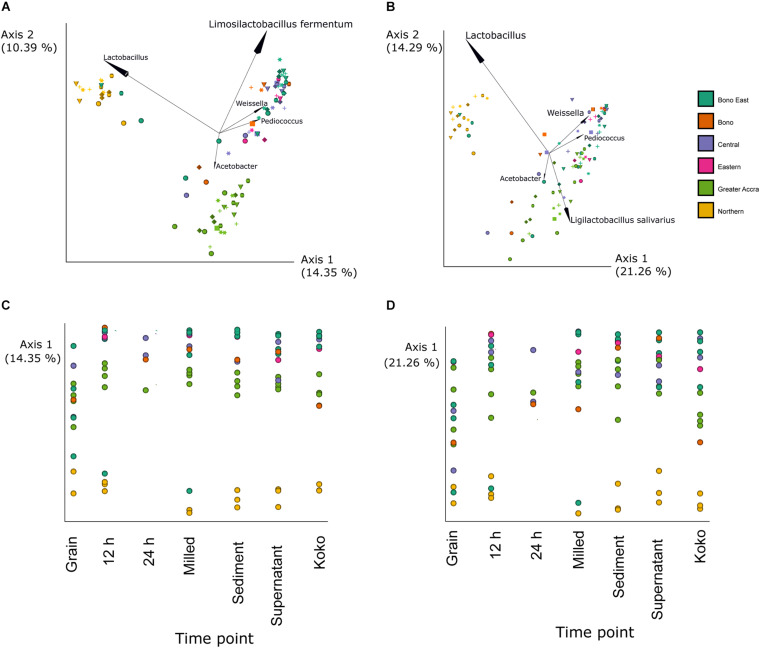
Bacterial beta diversity analysis. Principal coordinate analysis (PCoA) showing Jaccard **(A,C)** and unweighted UniFrac **(B,D)** distances. Samples produced in different regions are represented by different colors as indicated in the legend. In panels **(A,B)**, fermentation time points are represented by different shapes: grain (circle), milled (triangle), 12 h (hexagon), 24 h (square), sediment (cross), supernatant (star), and *Hausa koko* (diamond). The arrows indicate the five taxonomic groups (at genera or species level) that contribute most to the differences. In panels **(C,D)**, fermentation time points are represented along the *x*-axis. The percentage of variation explained by the plotted principal coordinates is indicated in the *y*-axis.

Changes in the bacterial populations through the fermentation process and between different regions were analyzed by principal coordinate analysis (PCoA) (Figure 3) based on Jaccard (Figures 3A,C) and unweighted Unifrac (Figures 3B,D) distances. Differences between groups were mainly caused by OTUs within the genera *Weissella*, *Pediococcus*, and *Acetobacter* as well as the genus *Lactobacillus* and the species *L. fermentum* and *L. salivarius*. For Jaccard distances, statistical differences were found between samples based on the region in which they were produced (*p*-value = 0.001). Pairwise comparison showed statistically significant differences between all regions except between Central and Bono or Eastern regions. For unweighted Unifrac distances, statistical differences were observed between samples based on the region of production (*p*-value = 0.001). Samples produced in Northern and Greater Accra regions were the only ones showing significant differences with all other regions (*p*-values Northern region vs.: Bono East = 0.001; Bono = 0.002; Central = 0.001; Eastern = 0.001; Greater Accra = 0.001; *p*-values Central vs.: Bono East = 0.001; Bono = 0.002; Central = 0.001; Eastern = 0.001; *p*-values Greater Accra region vs.: Bono East = 0.001; Bono = 0.001; Central = 0.001; Eastern = 0.002; Northern = 0.001). Statistically significant differences in unweighted Unifrac distances were also found between the different fermentation steps (*p*-value = 0.024). Particularly, grain samples were different from the rest of time points (*p*-value of grain samples vs.: 12 h = 0.001; milled = 0.005; supernatant = 0.002; sediment = 0.002; koko = 0.023), except for 24 h, although this could be caused by the smaller sample size of the 24-h group.

**FIGURE 4:**
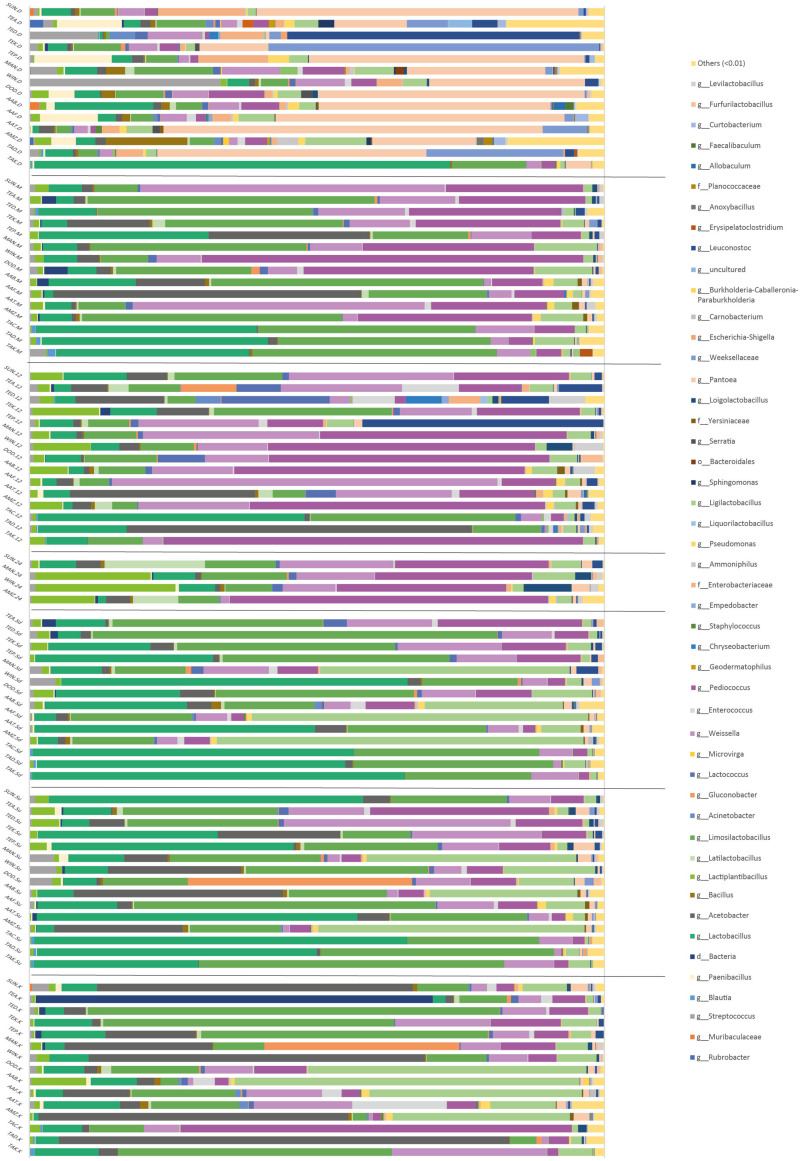
Relative abundance of the bacterial communities of the different processing steps during *Hausa koko* production. Each horizontal piled bar represents a single sample. Taxonomic groups at genera (g) level are identified where possible. Where genera could not be determined, order (o) or domain (d) are shown. Taxonomic groups with abundance <1% were included in the group “others.”

Over 400 different bacteria were profiled in this study. The samples from the different processors across the six regions showed that OTUs at the genus level in grain samples were mainly dominated by *Sphingomonas*, *Clostridium*, *Staphylococcus*, *Pseudomonas*, *Bacteroides*, *Chryseobacterium*, *Enterobacteriaceae*, and *Escherichia-Shigella*. Fermentation time points were, however, dominated by the genera *Lactobacillus* and *Acetobacter*. *Pediococcus*, *Weissella*, *Pantoea*, *Leuconostoc*, *Gluconobacter*, *Streptococcus*, and *Lactococcus* were also notable components. ANCOM at the genus level showed significant differences in the relative abundance depending on the processing step: the genus *Pantoea* (*W* = 842) decreased during the first fermentation step, and then was maintained at low levels, while the genus *Limosilactobacillus* increased during the fermentation process (*W* = 781) (Figure 4).

### Fungal Diversity

**FIGURE 5:**
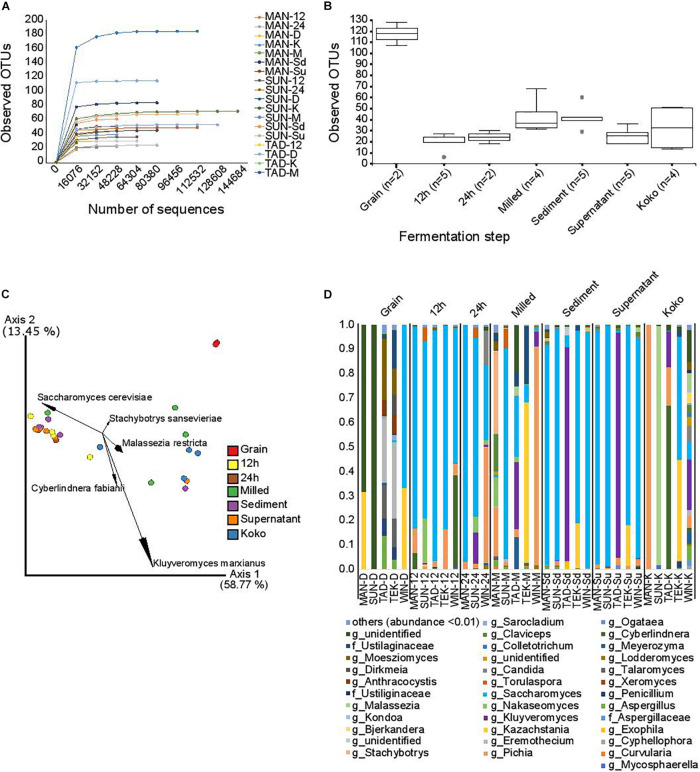
Fungal diversity. **(A)** Rarefaction plots of fungal observed OTUs depending on the sequencing depth. Each curve represents a sample. **(B)** Fungal alpha diversity based on the observed OTUs in the different fermentation steps. Dots represent outliers. **(C)** Fungal beta diversity represented by principal coordinate analysis based on weighted Unifrac distances. Fermentation time points are represented by different colors as indicated in the legend. The arrows indicate the five taxonomic groups that contribute most to the differences. **(D)** Relative abundance.

Fungal diversity was profiled in a subset of samples obtained from five of the processing locations. DNA sequencing of the ITS amplicons by the Illumina MiSeq platform resulted in 4,792,254 single-end sequence reads with an average of 145,219.8 ± 56,955.31 sequences per sample. Of these, 34.36% were discarded due to poor quality or after being identified as chimeras; as a result of these steps, 1,646,961 high-quality sequences were retained and analyzed, with an average of 49,907.90 ± 44,668.62 sequences per sample. As observed in Supplementary Table 2, the distribution of reads per sample was very variable. Prior to alpha and beta diversity analysis, the data were rarefied to 4,640 sequences per sample. Although not all the observed OTUs were captured (Figure 5A), this sampling depth enabled us to analyze most of the samples (all except SUN-D, MAN-D, WIN-D, WIN-M, WIN-24, and MAN-K). For alpha diversity indexes (Figure 5B), significant differences (*p*-value = 0.025) in observed OTUs were detected between groups based on fermentation time points. Although grain samples seem to contain more observed OTUs, differences were not statistically significant (*p* = 0.051), probably due to the small number of samples that could be retained after removing samples with less than 4,640 reads per sample. Pairwise comparison showed significant differences between the following fermentation stages: 12 h vs. milled (*p* = 0.014); 12 h vs. sediment (*p* = 0.009); grains vs. sediment (*p* = 0.051); grain vs. supernatant (*p* = 0.051); milled vs. supernatant (*p* = 0.049); and supernatant vs. sediment (*p* = 0.016).

Analysis of the beta diversity indexes showed significant differences (*p*-value = 0.001) for weighted Unifrac distance based on fermentation time points (Figure 5C). Pairwise comparison showed significant differences between the following fermentation time points: grain vs. 12 h (*p* = 0.039), grain vs. supernatant (*p* = 0.042), and 12 h vs. koko (*p* = 0.01). Differences in weighted Unifrac distances were caused mainly by OTUs within the species *Kluyveromyces marxianus*, *Saccharomyces cerevisiae*, *Cyberlindnera fabianii*, *Stachybotrys sansevieriae*, and *Malassezia restricta.*

Analysis of Composition of Microbiomes at the genus level showed significant differences in the relative abundance depending on the processing step: the genera *Saccharomyces* (*W* = 123) and *Pichia* (*W* = 111) were more abundant in samples after the fermentation started than in the grain samples. Figure 5D shows the relative abundance across the fermentation steps.

The grain samples were characterized by fungi of the genera *Kazachstania*, *Aspergillus*, *Penicillium*, *Talaromyces*, *Eremothecium*, *Anthracocystis*, *Moesziomyces*, *Sarocladium*, and *Dirkmeia*; a limited population of *Saccharomyces*; and a number of unidentified fungi. The 12-h and 24-h fermentation time points recorded a shift from this diverse population to a less diversified community, dominated by *Saccharomyces* and *Pichia. Candida*, *Kluyveromyces*, *Nakaseomyces*, *Torulaspora*, *Stachybotrys*, and *Cyberlindnera* were also present. The milled samples with spices also showed a diverse profile made up of OTUs including yeasts *Saccharomyces*, *Pichia*, *Kazachstania*, *Kluyveromyces*, and *Torulaspora*. The sediment and supernatant fermentation time points were mainly dominated by *Saccharomyces.* Other fungi such as *Kluyveromyces*, *Pichia*, *Kazachstania*, *Candida*, *Stachybotrys*, *Moesziomyces*, *Claviceps*, and *Malassezia* were also associated with this time point. *Hausa koko* samples were highly diversified with yeast communities dominated by *Pichia*, *Malassezia*, *Cyberlindnera*, *Kluyveromyces*, and *Saccharomyces.* The *Hausa koko* sample from Winneba particularly was highly diversified compared to the others. Other fungal genera identified included *Aspergillus*, *Meyerozyma*, *Eremothecium*, *Candida*, *Claviceps*, *Bjerkandera*, *Kondoa*, *Malassezia*, *Moesziomyces*, and some unidentified communities.

### Metabolic Composition

**FIGURE 6:**
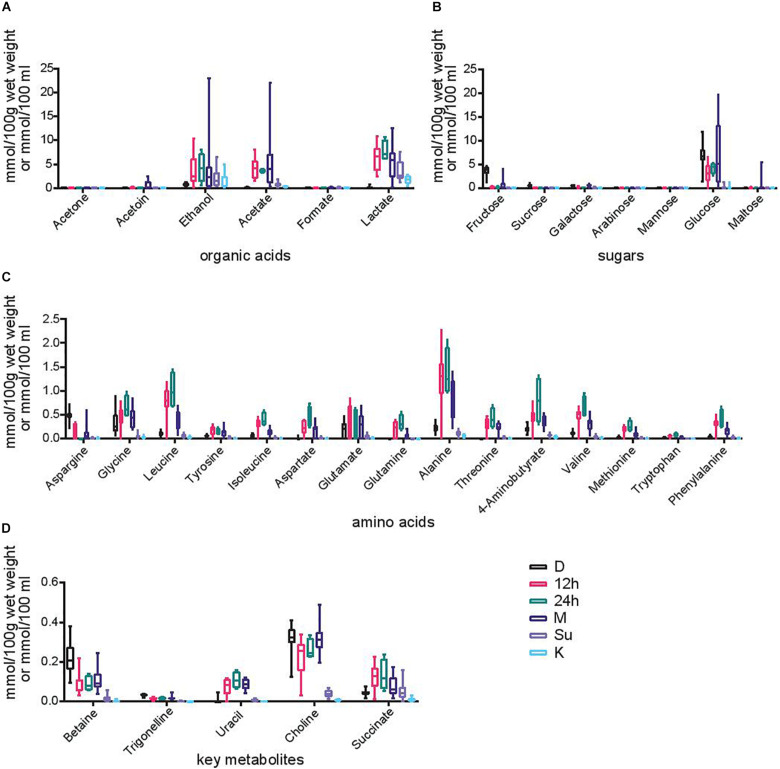
Metabolite profiles. Box plots of the concentration of various metabolites expressed in mmol per 100 g of wet weight for grains, 12 h, 24 h, and milled samples or mmol per 100 ml for supernatant and *Hausa koko* samples. Each box represents the 5th to 95th percentile. Each panel represents a different category of metabolites produced at different time points (dry, 12 h, 24 h, milled, supernatant, and koko) during *Hausa koko* production. **(A)** Organic acids, **(B)** sugars, **(C)** amino acids, and **(D)** key metabolites.

The metabolites present in the samples that are consumed (Su and K) as well as the different time points (D, 12 h, 24 h, and M) during *Hausa koko* production by 15 producers within six regions were analyzed. A total of 33 metabolites were detected and quantified (Figure 6) along the fermentation stages with varying trends in similar patterns in all the samples, irrespective of the geographical location the sample was obtained from. Two-way ANOVA test showed significant differences between time points with regard to organic acids. Among them, *post hoc* analysis showed significant increase in the concentration of lactate between grains and all the fermentation steps except *koko* (*p*-value grain vs. 12 h, 24 h, milled, and supernatant was <0.0001). Although no significant differences were detected, the concentration of lactate in *koko* was slightly higher than that in the grains. Similar changes were observed for acetate: statistical increases in acetate concentration between grains and 12 h (*p* < 0.0001), 24 h (*p* = 0.035), and milled samples (*p* < 0.0001) were detected. Again, statistical differences were found for ethanol between grains and 12 h (*p* < 0.0127), 24 h (*p* = 0.0342), and M (*p* < 0.0001). There were no significant differences for the other organic acids produced at the different time points. The changes observed in organic acids were inverse to the changes observed in sugar levels: glucose, the most abundant sugar found in the samples, decreased significantly in the first step of the fermentation (*p* < 0.0001) and increased slightly in the milled sample, although with a high variation between samples, to then decrease again in the supernatant and koko samples (*p* < 0.0001). Significant differences also existed in the concentration of fructose between grains and all the fermentation time points (*p* < 0.0001).

Another group of metabolites of importance identified at the various stages were amino acids (Figure 6C). The amino acid concentrations generally increased from the dry grains to the fermenting time points and reduced in the later stages of production, particularly in both supernatant and *Hausa koko* samples. Significant increase between grains and the 12- and 24-h fermentation points were detected for almost all of them (*p* < 0.0001 for leucine, isoleucine, aspartate, alanine, threonine, valine, and phenylalanine; *p* = 0.0007 for glutamate, *p* = 0.0002 for glutamine, and *p* = 0.0137 for methionine), although the concentration of glycine only increased significantly after 24 h (*p* = 0.0003) and asparagine decreased between these time points (*p* = 0.0001). Between the fermentation and milling of the samples, the concentration of some amino acids decreased significantly (*p* < 0.0001 for leucine, alanine, and valine; *p* = 0.0239 for glutamine), while for others, the decrease in concentration was observed in the supernatant or koko samples (*p* < 0.0001 for glycine, isoleucine, aspartate, alanine, threonine, and phenylalanine; *p* = 0.026 for tyrosine; *p* = 0.0005 for aspartate; *p* = 0.002 for glutamine; *p* = 0.02 for methionine).

Other metabolites not included in previous groups were detected at very low concentrations: betaine, trigonelline, uracil, choline, and succinate. Betaine and choline decreased after the first 12 and 24 h of fermentation (*p* < 0.0001) while uracil and succinate increased (*p* < 0.0001). The concentration of these compounds was maintained in the milled samples and dramatically decreased in supernatant and *koko* samples (*p* < 0.0001).

## Discussion

*Hausa koko* is one of the most popular fermented foods consumed in Ghana. It is an affordable and nutritious porridge for all people. Understanding the microbial community dynamics and the metabolic changes during the production of this fermented porridge is needed to design starter cultures to perform safer, standardized, and scalable production batches. In this study, we used culture-independent high-throughput sequencing of the 16S rRNA gene to study the bacterial diversity of *Hausa koko*, showing a high species richness at the different time points from the different geographical sampling locations. Significant differences were observed between the different time points during the production process.

The grain samples, used as the raw material to produce the *Hausa koko*, recorded more OTUs and higher relative abundance of the genus *Pantoea* compared to the other time points. This genus of Gram-negative bacteria of the Enterobacteriaceae family is usually isolated from several sources and ecological niches including plants (Kini et al., 2018; Azizi et al., 2020) and they are most probably associated with the original plant material and the soil environment (Sarita and Singh, 2016). The grain samples were also dominated by other Gram-negative microbes including *Pseudomonas*, *Enterobacteriaceae*, and *Escherichia-Shigella*, some of which may be potential pathogens and are commonly associated with feces, soil, and intestinal tracts of humans and warm-blooded animals and may cause gastroenteritis, diarrhea, vomiting, and nausea (Gadaga et al., 2004, 2008).

As expected, the fermentation stages presented a major shift to mainly fermentation-related genera dominated by lactic acid bacteria (LAB), which were detected already after 12 h of fermentation. A significant increase in the relative abundance of the genus *Limosilactobacillus* (formerly included in the *Lactobacillus* genus) (Zheng et al., 2020) was observed and other genera became dominant, including *Pediococcus*, *Weissella*, *Lactococcus*, *Streptococcus*, *Leuconostoc*, and other genera within the lactobacilli group, such as *Lactobacillus* and *Ligilactobacillus.* Consequently, an increase in the concentration of organic acids produced by the fermenting microbes was observed and a subsequent decrease in the population of *Pantoea* spp. was noticed. Shifts in the acidity after 24 to 48 h of fermentation of maize dough, used in the preparation of three popular Ghanaian traditional foods, have been reported (Halm et al., 2004). These organic acids, lactic acid in particular, are key fermentation features, important for both organoleptic qualities and pathogen reduction (Oguntoyinbo and Narbad, 2015). Sour foods are popular in Ghana, and in all such sour foods, the role of LAB has been demonstrated (Amoa-Awua et al., 1996; Obilie et al., 2004; Obodai and Dodd, 2006; Atter et al., 2014; Annan et al., 2015). The relative abundance of LAB populations increased along the fermentation stages from 12 to 24 h, M, Su, and Sd. The population, however, was reduced in the final porridge, which may be attributed primarily to application of heat. Acetic acid bacteria, *Acetobacter* and *Gluconobacter* genera, were also present in varying relative abundances at the different time points in the *Hausa koko* production process. Although not significant, some of the *Hausa koko* samples contained a higher relative abundance of acetic acid bacteria. Acetic acid bacteria produce mainly acetic acid, some vitamin C, and cellulose during spontaneous fermentation of foods and beverages (De Roos and De Vuyst, 2018). Their presence has been reported in other African cereal fermented foods including *kunu* and *burukutu* (Oguntoyinbo, 2014; Ezekiel et al., 2019). They are responsible for oxidation of ethanol produced during fermentation to acetic acid (Gómez-Manzo et al., 2010; Ezekiel et al., 2019).

Generally, significant differences did not occur between most of the samples within the same time point from the different regions or within different processing facilities of the same region, although some significant differences were detected in samples from the Northern and Greater Accra region, which could be attributed to the microbial communities of the millet substrates, as they may vary depending on the source. Additionally, the quality of water, spices, utensils, contact surfaces, and hands may influence these differences (Gadaga et al., 2008). The significant difference in the samples from the Northern region and the other five regions was due to OTUs within the genus *Lactobacillus* while the differences in the samples from the Greater Accra region seem to be attributed to OTUs within the *Acetobacter* genus. This study did not find any previous observations of regional differences in African cereal fermented foods. Therefore, further studies would be needed to determine what is causing those differences.

In this study, we selected a subset of five samples from three of the main regions, to characterize the fungal populations. Yeasts are the most common microorganisms apart from bacteria in spontaneous cereal fermentations (Achi and Ukwuru, 2015). Synergism occurs between bacteria and yeast in fermentation niches, with acidification of the medium by bacteria supporting yeast growth and subsequent release of amino acids and vitamins (Stadie et al., 2013). Factors such as the raw materials used, processing operations including duration and temperature of fermentation, hygienic practices, interactions between the microbes, and their successions, which are influenced by intrinsic and extrinsic growth factors, may influence the diversity of yeast in *Hausa koko* (Jespersen, 2003; Achi and Ukwuru, 2015; Johansen et al., 2019). The fungal community at the different stages of *Hausa koko* production was profiled using the ITS sequence data. Although most of the grain samples had to be removed in the diversity analysis due to the low number of reads obtained in the sample, the two samples that could be retained showed a highly diverse population of molds and yeast genera in the grains that are likely to be soil and grass inhabitants with very little *Saccharomyces*. In some of the grain samples, there was a high abundance of OTUs that could not be classified. The dominance of soil- and grass-associated fungi was drastically reduced in the fermentation time point samples at 12 h, 24 h, supernatants, and sediments. There was an increase in the relative abundance of the genera *Saccharomyces* and *Pichia.* Some of the samples were dominated by the genus *Kluyveromyces.* This trend may be attributed to acidification of the samples at these time points and microbial succession, which may have led to the inhibition of growth of some of these fungi while promoting the growth of others (Achi and Ukwuru, 2015). These time points were significantly different from the others due to OTUs classified as *S. cerevisiae*, which are predominant in indigenous African fermented cereal foods (Achi and Ukwuru, 2015; Johansen et al., 2019). The presence of *S. sansevieriae* and *M. restricta* in some of the milled samples have not been reported in indigenous African fermented foods yet. The genus *Pichia* is another frequently occurring yeast in fermented cereals and reported extensively. *Pichia kudriavzevii* was reported in another spontaneously fermented pearl millet product, *fura*, in West Africa (Pedersen et al., 2012), *ogi* (Omemu et al., 2007), and *gowé* (Greppi et al., 2013). *K. marxianus* is one of the promising yeast species isolated from fermented foods with beneficial characteristics and has been associated with spontaneous fermentation in West Africa (Karim et al., 2020; Motey et al., 2020). All the yeasts profiled at the different time points may contribute to production of aroma compounds from different carbon sources, mycotoxin degradation, increase in shelf life, safety, and nutritional value of *Hausa koko* (Forti et al., 2018; Johansen et al., 2019). Although significant reads from both bacteria and fungi were obtained from the K samples, cooking of the final *Hausa koko* porridge by addition of boiling water is expected to affect the number of live microbes in the final product. This could potentially reduce the level of most contaminants, potential pathogens, and aflatoxin-producing fungi that started the fermentation, making *Hausa koko* safer. It has been established that even though cooking of fermented cereal porridges actually reduces the antimicrobial effect of LAB and yeast on pathogens, significant inhibition of these pathogens still occurs (Mensah et al., 1991; Mensah, 1997).

The presence and interactions between the wide array of microbes at the various stages of *Hausa koko* production yielded different metabolites following the conversion of carbohydrates in accordance to the principles of fermentation (Hagman and Piškur, 2015). These metabolites were produced by the various fermenting microbes, predominantly LAB and yeast during this mixed fermentation process (Ohimain, 2016). Inconsistent trends in the sugar concentrations with a general increase in metabolic products of LAB and yeast was observed. There was a general reduction in the sugar concentrations along the processing time points. The milling breaks down the grains and makes the carbohydrates more accessible to the enzymes produced during fermentation. These enzymes then break down the complex carbohydrates into simple sugars that are utilized by the fermenting microbes for energy (mainly glycolysis). The spices may also have contributed to the increase in sugars. The high concentrations of sugars dominated by glucose in the milled samples make them ideal substrates and carbon source for use by fermenting microbes for growth (Charalampopoulos et al., 2002; Di Stefano et al., 2017). The production of organic acids such as lactate, acetate, and ethanol progressed steadily along the fermentation stages peaking in the milled millet samples. Dilution of the milled millet with water resulted in a decrease in their concentrations in the supernatants and *Hausa koko* samples. Other organic acids occurred in small concentrations. The presence of such organic metabolites is indicative of the involvement of heterofermentative LAB and yeast (Michodjèhoun-Mestres et al., 2005) and contributes to the flavor, taste, and sensorial properties of the final product (Sripriya et al., 1997; Onyango et al., 2000; Akpinar-Bayizit et al., 2010; Weldemichael et al., 2019). Amino acid concentrations in the grain samples were generally low but increased marginally in the 12-h fermented millet and milled samples (Mbithi-Mwikya et al., 2000; Adebiyi et al., 2017). This could be attributed to increased hydrolytic enzyme activities from the grains as well as breakdown of complex proteins to amino acids (Saleh et al., 2013). The concentration of some amino acids was reduced in the milled samples, but most of them decreased in the supernatant samples, which could be due to a combination of factors: the consumption by the microorganisms in order to multiply and the dilution with water in the supernatant and *Hausa koko* samples. Other important metabolites including betaine and trigonelline were profiled at the various time points of *Hausa koko* production. Whole grain cereals are excellent dietary sources for betaine and its precursor choline, which are associated with amino acid and lipid metabolism (Bruce et al., 2010; Ross et al., 2014). Trigonelline has been described as possessing anti-diabetic properties and decreasing blood cholesterol level, and is used for treating migraine, cancer, and other conditions (Basch et al., 2003; Bahmani et al., 2016).

The different metabolites profiled in the current study as a result of fermentation by the various microbes may contribute to the flavor and aroma of *Hausa koko*, similar to compounds developed during fermentation of other products (Salmerón et al., 2014; Weldemichael et al., 2019). These metabolites, including organic compounds, are antimicrobial substances involved in inhibiting the proliferation and survival of potential pathogens and other contaminants. Their presence contributes to the safety of the product and reduces incidence of diarrhea and other food borne diseases (Achi and Ukwuru, 2015; Adebo et al., 2017).

## Conclusion

Significant differences were observed in bacterial diversity at the different time points of *Hausa koko* production across the six regions. The grain samples were more diverse with high abundance of the genus *Pantoea*. Lactobacilli, however, increased steadily as fermentation progressed during the production stages, peaking in the sediment samples but reduced in the final *Hausa koko* as a result of dilution with water and application of heat. There were few differences in diversity between regions. The Northern region samples were different from the other regions due to OTUs within the genus *Lactobacillus*. ITS sequence data also revealed a high population of fungal genera in the grains including known contaminants, and soil and grass inhabitants with many unidentified. In general, the most abundant yeast during *Hausa koko* fermentation was the genus *Saccharomyces*. This may be the first time *S. sansevieriae* and *M. restricta* are reported in indigenous African fermented foods. *Hausa koko* samples recorded OTUs of the genera *Pichia*, *Malassezia*, *Cyberlindnera*, *Kluyveromyces*, and *Saccharomyces* and were differentiated from other time points by *C. fabianii* and *M. restricta.* The metabolomics study using NMR unveiled the profile of metabolites that were produced during *Hausa koko* fermentation. From the profile obtained, it can be suggested that traditional fermentation of *Hausa koko* undergoes the typical shifts from the fermentation process but the concentration of most of the organic acids and amino acids is drastically reduced in the final product that is consumed.

## Data Availability Statement

The sequencing datasets generated in this study can be found in the SRA database under the BioProject accession number PRJNA70037.

## Author Contributions

AA collected the samples, performed the DNA and metabolite extractions, interpreted the data, and drafted the original manuscript. MD supervised the work, performed bioinformatic and statistical analysis, interpreted the data, and contributed to the manuscript writing. KT-D and AK contributed to supervision and review and editing. MM and AN contributed to conceptualization, funding acquisition, supervision, and review and editing of the manuscript. IC performed nuclear magnetic resonance spectroscopy. DB sequenced the yeast samples. DN reviewed and edited the manuscript. WA-A contributed to conceptualization, supervision, and review and editing. All authors contributed to the article and approved the submitted version.

## Conflict of Interest

The authors declare that the research was conducted in the absence of any commercial or financial relationships that could be construed as a potential conflict of interest.

## Publisher’s Note

All claims expressed in this article are solely those of the authors and do not necessarily represent those of their affiliated organizations, or those of the publisher, the editors and the reviewers. Any product that may be evaluated in this article, or claim that may be made by its manufacturer, is not guaranteed or endorsed by the publisher.
